# Improved pregnancy outcomes with increasing antiretroviral coverage in South Africa

**DOI:** 10.1186/s12884-016-0821-3

**Published:** 2016-02-11

**Authors:** Theron Moodley, Dhayendre Moodley, Motshedisi Sebitloane, Niren Maharaj, Benn Sartorius

**Affiliations:** Department of Obstetrics and Gynaecology, School of Clinical Medicine, College of Health Sciences, University of KwaZulu Natal, Durban, South Africa; Womens Health and HIV Research Unit, Department of Obstetrics and Gynaecology, School of Clinical Medicine, College of Health Sciences, University of KwaZulu Natal, Durban, South Africa; Discipline of Public Health Medicine, School of Nursing and Public Health, College of Health Sciences, University of KwaZulu-Natal, Ground Floor, George Campbell Building, Durban, South Africa

## Abstract

**Background:**

Universal multi drug antiretroviral treatment in pregnancy is a global priority in our bid to eliminate paediatric HIV infections although few studies have documented the impact of antiretroviral coverage on overall pregnancy outcomes.

**Methods:**

We conducted a maternity audit at a large regional hospital in South Africa during July-December 2011 and January-June 2014 with an aim to determine an association between pregnancy outcomes and the ARV treatment guidelines implemented during those specific periods. During 2011, women received either Zidovudine/sd Nevirapine or Stavudine/Lamivudine/Nevirapine if CD4+ count was < 350 cells/ml. During 2014, all HIV positive pregnant women were eligible for a fixed dose combination (FDC) of triple ARVs (Tenofovir/Emtracitabine/Efavirenz).

**Results:**

In 2011, 622 (35.9 %) of 1732 HIV positive pregnant women received triple antiretrovirals (D4T/3TC/NVP) and in 2014, 2104 (94.8 %) of 2219 HIV positive pregnant women received the fixed dose combination (TDF/FTC/EFV). We observed a reduction in the proportion of unregistered pregnancies, caesarean delivery rate, still birth rate, very low birth weight rate, and very premature delivery rate in 2014. In a bivariate analysis of all 9,847 deliveries, unregistered pregnancies (2.2 %) and HIV infection (37.8 %) remained significant risk factors for SB(OR 6.36 and 1.43 respectively), PTD(OR 4.23 and 1.26 respectively),LBW (OR 4.07 and 1.26 respectively) and SGA(OR 2.17 and 1.151 respectively). In a multivariable analysis of HIV positive women only, having received AZT/NVP or D4T/3TC/NVP or EFV/TDF/FTC as opposed to not receiving any ARV was significantly associated with reduced odds of a SB (OR 0.08, 0.21 and 0.18 respectively), PTD (OR 0.52, 0.68 and 0.56 respectively) and LBW(0.37, 0.61 and 0.52 respectively).

**Conclusion:**

An improvement in birth outcomes is likely associated with the increased coverage of triple antiretroviral treatment for pregnant women. And untreated HIV infected women and women who do not seek antenatal care should be considered most at risk for poor birth outcomes.

## Background

Since 2005, like most sub Saharan African countries, South Africa prioritised antiretroviral therapy (ART) for HIV positive pregnant women to reduce the risk of mother-to-child transmission (MTCT) and improve maternal health [1]. Over the years, the World Health Organisation (WHO) recommended longer duration and multi-drug antiretroviral combinations with improved efficacy in preventing MTCT (PMTCT), and eligibility for lifelong antiretroviral treatment for pregnant women was extended resulting in a remarkable reduction in the perinatal transmission rate at 6 weeks and an improvement in HIV free-survival of children under 2 years of age [2–4]. More recently, WHO recommendations changed yet again to improve coverage of antiretroviral treatment of women deserving more than just PMTCT prophylaxis [5]. To ensure that women needing antiretroviral treatment are commenced early in pregnancy, sub-Saharan African (SSA) countries most affected by the HIV pandemic adopted the World Health Organisation’s recommendations that all HIV positive women should be commenced on a fixed dose combination (FDC) triple antiretroviral combination (Tenofovir 300 mg, Emtricitabine 200 mg and Efavirenz 600 mg) irrespective of immunological status or disease stage [6]. These progressive changes in HIV management guidelines are intended to address the Millenium Development Goals (MDG) 4 (reduce child mortality), 5 (improve maternal health) and 6 (combat HIV/AIDS) [7]. While substantial progress has been made in reducing the under 5 child and maternal mortality globally, certain regions such as sub-saharan Africa and Southern Asia still need to accelerate progress.

Antiretroviral treatment for women in their reproductive years is expected to have the greatest impact in reducing child mortality associated with HIV infection. However few countries are expected to achieve the MDG 4 target by 2015 [8]. While the under 5 mortality has been reduced by a third since 2000, almost half of the deaths were attributed to infectious causes and 44 % of deaths occurred in the neonatal period [9]. The leading causes of neonatal death were preterm birth complications, intrapartum-related complications, congenital defects incompatible with life and sepsis.

Early studies on use of antiretrovirals in pregnancy in Europe and the US have drawn conflicting associations between antiretrovirals and birth outcomes particularly preterm delivery, low birth weight and still births [10–12]. While clinical trial findings may suggest that the benefits of antiretroviral use in pregnancy far outweigh the risk of adverse birth outcomes the changing trend in child mortality particularly neonatal deaths in high HIV burdened countries alludes to the possible association between HIV and antiretroviral use and adverse birth outcomes [9]. Studies in subSaharan Africa have produced conflicting evidence. In early studies before the full scale introduction of antiretroviral treatment in Malawi and Tanzania, HIV disease severity as defined by high viral load or low CD4+ count was significantly associated with preterm delivery and/or low birth weight [13, 14] . In Botswana, stillbirth, preterm delivery (PTD), small for gestational age (SGA), and neonatal deaths were shown to be higher in HIV positive women in comparison to their HIV negative counterparts, but the introduction of HAART increased the risk further [15]. Moreover, the recent findings of a multinational PMTCT study suggest an increased risk of preterm deliveries and low birth weight with a triple ARV regimen as compared to a Zidovudine based dual regimen. The study further reported a higher incidence of more severe birth outcomes (PTD <34w) associated with a Truvada based triple regimen as compared to the Combivir based triple regimen [16].

To determine if antiretroviral coverage and antiretroviral regimens influence overall pregnancy outcomes we conducted a maternity audit during two defined periods of implementing new ART guidelines in a South African population where one in three pregnant women are HIV infected.

## Methods

We conducted a cross sectional analysis of birth data abstracted from maternity registers of a regional hospital in Durban, South Africa for the period July to December 2011 and January to June 2014. This retrospective data analysis included all women with viable pregnancies delivering a neonate greater than or equal to 500 g and whose birth outcomes were recorded in the maternity register. Women who had a multiple birth would naturally affect preterm birth and birth weight outcomes and were thus excluded from the analyses i.e. 160 twin pregnancies and 3 triplet pregnancies were excluded. For the period July - December 2011 HIV positive women were receiving dual ARV prophylaxis [Zidovudine(ZDV) from 14 weeks in pregnancy + single dose Nevirapine (NVP)in labour/delivery] or if eligible for ARV treatment (CD4+ count <350) pregnant women would have initiated a triple antiretroviral regimen [Stavudine (D4T) + Lamivudine (3TC) + Nevirapine (NVP)]. For the period January to June 2014 all HIV positive pregnant women were eligible for a fixed dose combination (FDC) of triple ARVs [Tenofovir (TDF) + Emtracitabine (FTC) + Efavirenz (EFV)] independent of CD4+ count.

The regional hospital supports 17 primary health care clinics and has an annual birth rate of 12,000. Women receive antenatal care at the primary health care clinics that have recorded an estimated HIV antenatal prevalence of 40 %.

### Study variables

The maternity register is routinely completed by the midwife immediately after a delivery. The midwife would transcribe maternal data such as age, HIV status, CD4+ count, ARV regimen and whether women registered for antenatal care from a patient-held antenatal record onto the maternity register. Antiretroviral regimens were recorded as “Dual” or “Triple” or “Fixed Dose Combination (FDC)”. Each of these has previously been described in greater detail. Women who had no record of antenatal care prior to hospital admission were defined as unregistered pregnancies. Other data recorded by the midwife include the mode of delivery, Live birth/Still birth, Birth Weight, and infant’s gestational age at delivery based on mothers last normal menstrual period or ultrasound data or a combination of both. For our analysis, a premature birth was defined as a birth <37 weeks of gestation and low birth weight was defined as a newborn weighing < 2500 grams. In considering severity these variables were further subcategorized into moderate-to-severe premature birth if gestational age at birth <34 weeks and very low birth weight if newborn weight < 1500 grams. The mode of delivery at public hospitals in South Africa is usually guided by the woman’s obstetric history, current pregnancy complications and progress in labour. For the former two criteria, a ceasarean delivery is planned and poor progress in labour or complications in labour usually lead to an emergency ceasarean delivery. In all other cases the mode of delivery is documented as Vaginal.

A complete dataset used in this analysis is stored on site and available upon request.

### Statistical analysis

The maternity registers for the specific study periods were captured onto an excel spreadsheet. Data were analyzed using Stata 13.0 (StataCorp (2013) Stata Statistical Software: Release 13. College Station, TX: StataCorp LP). Birth outcomes were dichotomized and point estimates along with associated 95 % binomial confidence intervals were calculated. Significant association between categorical outcomes and categorical explanatory variables (e.g. birth outcome versus maternal HIV disease stage) were assessed using a Pearson chi-square (*χ*^2^) test. If an expected cell count in the contingency tables had fewer than 5 observations then the Fisher’s exact test was used instead. Continuous explanatory (independent) variables were compared across dichotomous or multiple (≥3 groups) birth outcomes groups using a *t*-test and one way analysis of variance (ANOVA) respectively. If the data were not normal then non-parametric equivalents, namely the Wilcoxon rank-sum and Kruskal Wallis test respectively, were used. Odds ratios (OR) were calculated using a bivariate and multivariable adjusted logistic regression to adjust for potential confounding. We clustered on mother to account for multiple measurements within the mother cluster i.e. for those women who had had more than one distinct pregnancy episode in the study period. Both a self-selection and stepwise approach for multivariable model building were compared. Final model fit and adequacy (goodness-of-fit) were also assessed. An adjusted p-value of <0.05 was deemed statistically significant.

Institutional Regulatory approval was obtained from the University of KwaZulu Natal Biomedical Research Ethics Committee. All patient details for the study were maintained as confidential and unique study participant numbers replaced in-patient’s hospital numbers to conceal participant identity. This being a maternity audit, patient information extracted from maternity registers were anonymized and de-identified prior to analysis.

## Results

There were 9847deliveries included in this analysis [after exclusions of twin (or multiple gestation pregnancies) and pregnancies with missing outcomes], 4369 (44.4 %) of whom occurred in 2011 and 5479 (55.6 %) occurred in 2014. Of the total 9847women, 200 (2.0 %) women did not seek antenatal care and 3723 (37.8 %) of women who sought antenatal care were HIV positive. Forty two percent (507/1199) of the HIV positive women had a reported CD4+ count < 350 cells/mm^3^ in pregnancy, 148 (4 %) did not receive any ARV, 974 (26.2 %) received dual ARV prophylaxis and 2573 (69.1 %) received triple ARV treatment.

When compared to 2011, we observed a reduction in the proportion of unregistered pregnancies, caesarean delivery rate, still birth rate, very low birth weight rate, and very premature delivery rate in 2014 (Table 1). In 2011, 576 (35.6 %) of 1618 HIV positive pregnant women received triple antiretrovirals (D4T/3TC/NVP) and in 2014, 1997 (94.9 %) of 2105 HIV positive pregnant women received the fixed dose combination (TDF/FTC/EFV). Overall, among women who had a CD4+ count result by delivery, we reported the highest Very Low Birth Weight (VLBW)(222/1000 births), Low Birth Weight (LBW) (667/1000 births), Still Birth (SB) (333/1000 births), and Preterm Delivery (PTD)(666/1000 births) rates among HIV positive women with a CD4 + <350 and not receiving any ARV. However, due to the small number of women with a CD4,<350 (*n* = 9), these rates should be treated with caution. Of note, significantly lower rates of SB, LBW, VLBW, and PTD rates were observed for HIV positive women receiving some form of ARV prophylaxis or treatment independent of the CD4+ count. Unregistered pregnancies, similar to HIV positive women not receiving any ARV, resulted in significantly higher LBW, VLBW, SBR, PTD and Very Preterm Delivery (VPTDR) (335/100, 185/1000, 125/1000, 490 and 225/1000 births respectively) than any of the other categories.

**Table 1:** Stillbirth outcome overall and for HIV + ve only

All women						
				Bivariate	Multivariable ^iv^	
Variable	Live births: n (% ^i^)	Stillbirths: n (%)	*p*-value ^ii^	0R ^iii^ (95 % CI)	0R (95 % CI)	Adj. *p*-value
Year						
2011	4226 (96.73)	143 (3.27)	0.006	1 (ref)	1 (ref)	
2014	5349 (97.65)	129 (2.35)		0.71 (0.56–0.91)	0.74 (0.56–0.97)	0.03
Age Category						
< 18	812 (98.19)	15 (1.81)	0.045	1 (ref)	1 (ref)	
18–24	4248 (97.52)	108 (2.48)		1.38 (0.8–2.37)	1.37 (0.79–2.38)	0.26
25–34	3544 (96.78)	118 (3.22)		1.8 (1.05–3.1)	1.86 (1.06–3.25)	0.03
> 34	863 (96.64)	30 (3.36)		1.88 (1.01–3.52)	1.99 (1.04–3.81)	0.037
Mode of Delivery						
Vaginal	6480 (96.95)	204 (3.05)	0.006	1 (ref)	1 (ref)	
Emergency Caesarean	1420 (97.46)	37 (2.54)		0.83 (0.58–1.18)	0.71 (0.48–1.04)	0.08
Planned Caesarean	1661 (98.34)	28 (1.66)		0.54 (0.36–0.8)	0.58 (0.39–0.88)	0.01
HIV						
Negative	5786 (97.8)	130 (2.2)	<0.001	1 (ref)	1 (ref)	
Positive	3607 (96.88)	116 (3.12)		1.43 (1.11–1.84)	1.26 (0.97–1.65)	0.088
Unregistered Antenatal	175 (87.5)	25 (12.5)		6.36 (4.04–10.01)	6.1 (3.86–9.64)	<0.001
HIV + ve women only						
				Bivariate	Multivariable ^iv^	
Variable	Live births: n (%)	Stillbirths: n (%)	*p*-value	0R (95 % CI)	0R (95 % CI)	Adj. *p*-value
Year						
2011	1559 (96.35)	59 (3.65)	0.102	1 (ref)	1 (ref)	
2014	2048 (97.29)	57 (2.71)		0.74 (0.51–1.06)	0.52 (0.27–0.97)	0.041
Age Category						
< 18	89 (95.7)	4 (4.3)	0.018	1 (ref)	1 (ref)	
18–24	1167 (98.15)	22 (1.85)		0.42 (0.14–1.24)	0.79 (0.18–3.5)	0.759
25–34	1830 (96.16)	73 (3.84)		0.89 (0.32–2.48)	1.71 (0.41–7.24)	0.463
> 34	488 (96.83)	16 (3.17)		0.73 (0.24–2.23)	1.41 (0.31–6.4)	0.66
Mode of Delivery						
Vaginal	2385 (96.52)	86 (3.48)	0.093	1 (ref)	1 (ref)	
Emergency Caesarean	548 (97.34)	15 (2.66)		0.76 (0.44–1.32)	0.75 (0.4–1.39)	0.362
Planned Caesarean	666 (98.09)	13 (1.91)		0.54 (0.3–0.98)	0.57 (0.31–1.06)	0.074
CD4 category						
> 500	333 (97.65)	8 (2.35)	0.047	1 (ref)	Excluded as co-linear with ART regimen	
351–500	341 (99.42)	2 (0.58)		0.24 (0.05–1.16)		
201–350	327 (96.75)	11 (3.25)		1.4 (0.56–3.53)		
< 200	169 (96.02)	7 (3.98)		1.72 (0.61–4.83)		
ART regimen						
Nil ARVs	128 (86.49)	20 (13.51)	<0.001	1 (ref)	1 (ref)	
AZT/NVP	957 (98.25)	17 (1.75)		0.11 (0.06–0.22)	0.08 (0.04–0.16)	<0.001
D4T/3TC/NVP	869 (95.81)	38 (4.19)		0.28 (0.16–0.5)	0.2 (0.11–0.38)	<0.001
EFV/TDF/FTC	1629 (97.78)	37 (2.22)		0.15 (0.08–0.26)	0.18 (0.1–0.34)	<0.001

*i* row percentage, *ii* Chi-square, *iii* Odds Ratio, *iv* following variables were adjusted for in the multivariable adjusted model, *year* age group, mode of delivery, pregnancy term category, HIV, status

*i* AZT/NVP vs D4T/3TC/NVP p-value = 0.002, *ii* AZT/NVP vs FTC p-value = 0.041, *iii* D4T/3TC/NVP vs FTC p-value = 0.768, *iv* following variables were adjusted for in the multivariable adjusted model, *year* age group, mode of delivery, pregnancy term category, HIV status, CD4 (HIV positive mothers only), ART regimen (HIV positive mothers only)

### Still births

Table 1 displays the results of a multivariable analysis of all women to identify the adjusted ORs and their 95 % confidence intervals for birth outcomes. Older maternal age categories, namely 25–34 and > 34 (OR 1.86 95 CI: 1.06–3.25 and 1.99 95 % CI: 1.04–3.81 respectively) and unregistered pregnancies (OR 6.51; 95 % CI: 3.86–9.64) remained significant risk factors for still birth among all subjects. Later year of delivery (2014) (OR 0.74; 95 CI: 0.56–0.97) and planned caesarean delivery (OR 0.58; 95 % CI: 0.39–0.88) were significantly associated with reduced odds of stillbirth.

Table 1 also displays the findings of a multivariable analysis for HIV positive women only. Having received any one of the ARV regimens (AZT/NVP or D4T/3TC/NVP or EFV/TDF/FTC) was significantly associated with a highly reduced odds of a stillbirth (ORs of 0.08, 0.20, 0.18 respectively) in comparison to women who did not receive any ARV. Pairwise comparison suggested that the AZT/NVP regimen was significantly better than D4T/3TC/NVP and EFV/TDF/FTC while other pairwise comparisons of the 3 antiretroviral regimens did not yield further significant differences.

### Preterm deliveries, Low birth weight and small for gestational Age

After a multivariable analysis, HIV infection (OR’s ~ 1.3), emergency c-section and unregistered pregnancies (OR’s ~ 4.2) remained strongly associated with both pre-term delivery (Table 2) and low birth weight (Table 3) respectively. Later year of delivery was associated with an increased odds of LBW (OR = 1.3). Similarly later year of delivery (OR = 1.21), and unregistered pregnancy (OR 2.28) were also significant risk factors for small for gestational age (GA) following multivariable adjustment (Table 4).

**Table 2:** Pre-term outcome overall and for HIV + ve only

All women				Bivariate	Multivariable ^iv^	
Variable	Term n (% ^i^)	Pre-term <37w) n (%)	*p*-value ^ii^	0R ^iii^ (95 % CI)	0R (95 % CI)	adj *p*-value
Year						
2011	3409 (78.03)	960 (21.97)	0.002	1 (ref)	1 (ref)	
2014	4412 (80.54)	1066 (19.46)		0.86 (0.78–0.95)	0.94 (0.83–1.06)	0.291
Age Category						
< 18	643 (77.75)	184 (22.25)	0.053	1 (ref)	1 (ref)	
18–24	3421 (78.54)	935 (21.46)		0.96 (0.8–1.14)	0.92 (0.77–1.1)	0.362
25–34	2945 (80.42)	717 (19.58)		0.85 (0.71–1.02)	0.78 (0.64–0.94)	0.01
> 34	726 (81.3)	167 (18.7)		0.8 (0.64–1.02)	0.73 (0.57–0.93)	0.012
Mode of Delivery						
Vaginal	5365 (80.27)	1319 (19.73)	<0.001	1 (ref)	1 (ref)	
Emergency Caesarean	1100 (75.5)	357 (24.5)		1.32 (1.15–1.51)	1.3 (1.12–1.51)	0.001
Planned Caesarean	1346 (79.69)	343 (20.31)		1.04 (0.91–1.18)	1.07 (0.93–1.24)	0.319
HIV						
Negative	4820 (81.47)	1096 (18.53)	<0.001	1 (ref)	1 (ref)	
Positive	2893 (77.71)	830 (22.29)		1.26 (1.14–1.4)	1.33 (1.19–1.48)	<0.001
Unregistered Antenatal	102 (51)	98 (49)		4.23 (3.18–5.62)	4.24 (3.18–5.64)	<0.001
HIV + ve women only				Bivariate	Multivariable ^v^	
Variable	Term n (% ^i^)	Pre-term (<37w) n (%)	*p*-value ^ii^	0R (95 % CI)	0R (95 % CI)	adj *p*-value
Year						
2011	1249 (77.19)	369 (22.81)	0.51	1 (ref)	1 (ref)	
2014	1644 (78.1)	461 (21.9)		0.95 (0.81–1.11)	0.63 (0.27–1.44)	0.269
Age Category						
< 18	69 (74.19)	24 (25.81)	0.849	1 (ref)	1 (ref)	
18–24	925 (77.8)	264 (22.2)		0.82 (0.51–1.33)	1.14 (0.32–4.01)	0.84
25–34	1482 (77.88)	421 (22.12)		0.82 (0.51–1.32)	1.26 (0.36–4.4)	0.717
> 34	395 (78.37)	109 (21.63)		0.79 (0.48–1.32)	1.35 (0.36–5)	0.655
Mode of Delivery						
Vaginal	1940 (78.51)	531 (21.49)	0.13	1 (ref)	1 (ref)	
Emergency Caesarean	420 (74.6)	143 (25.4)		1.24 (1.01–1.54)	1.43 (1–2.06)	0.05
Planned Caesarean	526 (77.47)	153 (22.53)		1.06 (0.87–1.3)	0.82 (0.49–1.37)	0.456
CD4 category						
< 200	274 (80.35)	67 (19.65)	0.582	1 (ref)	1 (ref)	
201–350	282 (82.22)	61 (17.78)		0.88 (0.6–1.3)	0.87 (0.58–1.29)	0.478
351–500	275 (81.36)	63 (18.64)		0.94 (0.64–1.37)	0.92 (0.6–1.39)	0.686
> 500	136 (77.27)	40 (22.73)		1.2 (0.77–1.87)	1.16 (0.72–1.88)	0.545
ART regimen						
Nil ARVs	100 (67.57)	48 (32.43)	0.002	1 (ref)	1 (ref)	
AZT/NVP	778 (79.88)	196 (20.12)		0.52 (0.36–0.77)	0.2 (0.08–0.51)	0.001
D4T/3TC/NVP	685 (75.52)	222 (24.48)		0.68 (0.46–0.98)	0.21 (0.08–0.55)	0.001
EFV/TDF/FTC	1315 (78.93)	351 (21.07)		0.56 (0.39–0.8)	0.31 (0.11–0.9)	0.031

*i* row percentage, *ii* Chi-square, *iii* Odds Ratio, *iv* following variables were adjusted for in the multivariable adjusted model, *year* age group, mode of delivery, pregnancy term category, HIV status

*i* Chi-square, *ii* AZT/NVP vs D4T/3TC/NVP p-value = 0.714, *iii* AZT/NVP vs FTC p-value = 0.321, *iv* D4T/3TC/NVP vs FTC p-value = 0.368, *v* following variables were adjusted for in the multivariable adjusted model, *year* age group, mode of delivery, pregnancy term category, HIV status, CD4 (HIV positive mothers only), ART regimen (HIV positive mothers only)

**Table 3:** Low Birth Weight overall and for HIV + ve only

All women						
				Bivariate	Multivariable ^v^	
Variable	Normal BW ^i^ (≥2500 g): n (% ^ii^)	LBW (<2500 g): n (%)	*p*-value ^iii^	0R ^iv^ (95 % CI)	0R (95 % CI)	Adj. *p*-value
Year						
2011	3846 (88.03)	523 (11.97)	0.238	1 (ref)	1 (ref)	
2014	4779 (87.24)	699 (12.76)		1.08 (0.95–1.21)	1.26 (1.08–1.46)	0.003
Age Category						
< 18	717 (86.7)	110 (13.3)	0.82	1 (ref)	1 (ref)	
18–24	3825 (87.81)	531 (12.19)		0.9 (0.73–1.13)	0.85 (0.68–1.07)	0.166
25–34	3203 (87.47)	459 (12.53)		0.93 (0.75–1.17)	0.84 (0.66–1.06)	0.134
> 34	779 (87.23)	114 (12.77)		0.95 (0.72–1.26)	0.85 (0.63–1.13)	0.264
Mode of Delivery						
Vaginal	5941 (88.88)	743 (11.12)	<0.001	1 (ref)	1 (ref)	
Emergency Caesarean	1230 (84.42)	227 (15.58)		1.48 (1.26–1.73)	1.72 (1.42–2.07)	<0.001
Planned Caesarean	1440 (85.26)	249 (14.74)		1.38 (1.18–1.61)	1.32 (1.12–1.55)	0.001
HIV						
Negative	5265 (89)	651 (11)	<0.001	1 (ref)	1 (ref)	
Positive	3221 (86.52)	502 (13.48)		1.26 (1.11–1.43)	1.26 (1.11–1.44)	0.001
Unbooked	133 (66.5)	67 (33.5)		4.07 (3–5.53)	4.28 (3.15–5.82)	<0.001
HIV + ve women only						
				Bivariate	Multivariable ^iv^	
Variable	Normal BW (≥2500 g) : n (%)	LBW (<2500 g): n (%)	*p*-value	0R (95 % CI)	0R (95 % CI)	Adj. *p*-value
Year						
2011	1416 (87.52)	202 (12.48)	0.118	1 (ref)	1 (ref)	
2014	1805 (85.75)	300 (14.25)		1.17 (0.96–1.41)	0.65 (0.24–1.79)	0.407
Age Category						
< 18	80 (86.02)	13 (13.98)	0.187	1 (ref)	1 (ref)	
18–24	1050 (88.31)	139 (11.69)		0.81 (0.44–1.5)	1.68 (0.22–13.06)	0.618
25–34	1631 (85.71)	272 (14.29)		1.03 (0.56–1.87)	2.11 (0.28–16.2)	0.472
> 34	431 (85.52)	73 (14.48)		1.04 (0.55–1.97)	1.84 (0.22–15.15)	0.569
Mode of Delivery						
Vaginal	2177 (88.1)	294 (11.9)	<0.001	1 (ref)	1 (ref)	
Emergency Caesarean	472 (83.84)	91 (16.16)		1.43 (1.11–1.84)	2.09 (1.32–3.31)	0.002
Planned Caesarean	564 (83.06)	115 (16.94)		1.51 (1.19–1.91)	1.19 (0.64–2.21)	0.58
CD4 category						
> 500	300 (87.98)	41 (12.02)	0.095	1 (ref)	1 (ref)	
351–500	317 (92.42)	26 (7.58)		0.6 (0.36–1.01)	0.54 (0.31–0.92)	0.025
201–350	300 (88.76)	38 (11.24)		0.93 (0.58–1.48)	0.91 (0.54–1.52)	0.718
< 200	151 (85.8)	25 (14.2)		1.21 (0.71–2.07)	0.94 (0.51–1.72)	0.84
ART regimen						
Nil ARVs	114 (77.03)	34 (22.97)	<0.001	1 (ref)	1 (ref)	
AZT/NVP i, ii	877 (90.04)	97 (9.96)		0.37 (0.24–0.57)	0.06 (0.02–0.18)	<0.001
D4T/3TC/NVP iii	768 (84.67)	139 (15.33)		0.61 (0.4–0.93)	0.09 (0.03–0.24)	<0.001
EFV/TDF/FTC	1442 (86.55)	224 (13.45)		0.52 (0.35–0.78)	0.12 (0.04–0.37)	<0.001

*i* Birth weight (BW), *ii* row percentage, *iii* Chi-square, *iv* Odds Ratio, *v* following variables were adjusted for in the multivariable adjusted model, *year* age group, mode of delivery, pregnancy term category, HIV status

*i* AZT/NVP vs D4T/3TC/NVP p-value = 0.219, *ii* AZT/NVP vs FTC p-value = 0.287, *iii* D4T/3TC/NVP vs FTC p-value = 0.594, *iv* following variables were adjusted for in the multivariable adjusted model, *year* age group, mode of delivery, pregnancy term category, HIV status, CD4 (HIV positive mothers only), ART regimen (HIV positive mothers only)

**Table 4:** Small for gestational age overall and for HIV + ve only

All women						
				Bivariate	Multivariable vi	
Variable	Normal for GA ^i^: n (% ^ii^)	Small for GA ^iii^: n (%)	*p*-value ^iv^	0R ^v^ (95 % CI)	0R (95 % CI)	Adj. *p*-value
Year						
2011	4053 (92.77)	316 (7.23)	0.066	1 (ref)	1 (ref)	
2014	5027 (91.77)	451 (8.23)		1.15 (0.99–1.34)	1.21 (1.02–1.44)	0.032
Age Category						
< 18	755 (91.29)	72 (8.71)	0.106	1 (ref)	1 (ref)	
18–24	4032 (92.56)	324 (7.44)		0.84 (0.65–1.1)	0.82 (0.63–1.08)	0.161
25–34	3384 (92.41)	278 (7.59)		0.86 (0.66–1.13)	0.81 (0.61–1.07)	0.137
> 34	807 (90.37)	86 (9.63)		1.12 (0.8–1.55)	1.06 (0.75–1.48)	0.750
Mode of Delivery						
Vaginal	6180 (92.46)	504 (7.54)	0.300	1 (ref)	1 (ref)	
Emergency Caesarean	1340 (91.97)	117 (8.03)		1.07 (0.87–1.32)	1.21 (0.95–1.53)	0.124
Planned Caesarean	1543 (91.36)	146 (8.64)		1.16 (0.96–1.41)	1.11 (0.91–1.35)	0.288
HIV						
Negative	5487 (92.75)	429 (7.25)	<0.001	1 (ref)	1 (ref)	
Positive	3417 (91.78)	306 (8.22)		1.15 (0.98–1.33)	1.15 (0.98–1.35)	0.078
Unbooked	171 (85.5)	29 (14.5)		2.17 (1.45–3.25)	2.28 (1.5–3.45)	<0.001
HIV + ve women only						
				Bivariate	Multivariable iv	
Variable	Normal for GA: n (% ^ii^)	Small for GA: n (%)	*p*-value	0R (95 % CI)	0R (95 % CI)	Adj. *p*-value
Year						
2011	1491 (92.15)	127 (7.85)	0.471	1 (ref)	1 (ref)	
2014	1926 (91.5)	179 (8.5)		1.09 (0.86–1.38)	1.53 (0.51–4.61)	0.447
Age Category						
< 18	84 (90.32)	9 (9.68)	0.048	1 (ref)	1 (ref)	
18–24	1110 (93.36)	79 (6.64)		0.66 (0.32–1.37)	0.67 (0.14–3.16)	0.609
25–34	1741 (91.49)	162 (8.51)		0.87 (0.43–1.76)	0.80 (0.17–3.75)	0.778
> 34	451 (89.48)	53 (10.52)		1.10 (0.52–2.31)	1.27 (0.26–6.28)	0.771
Mode of Delivery						
Vaginal	2282 (92.35)	189 (7.65)	0.176	1 (ref)	1 (ref)	
Emergency Caesarean	509 (90.41)	54 (9.59)		1.28 (0.93–1.76)	1.64 (0.98–2.74)	0.059
Planned Caesarean	616 (90.72)	63 (9.28)		1.23 (0.92–1.66)	0.82 (0.40–1.68)	0.584
CD4 category						
> 500	312 (91.5)	29 (8.5)	0.583	1 (ref)	1 (ref)	
351–500	321 (93.59)	22 (6.41)		0.74 (0.41–1.31)	0.72 (0.40–1.29)	0.267
201–350	307 (90.83)	31 (9.17)		1.09 (0.64–1.85)	1.20 (0.69–2.09)	0.520
< 200	161 (91.48)	15 (8.52)		1.00 (0.52–1.92)	0.94 (0.45–1.94)	0.862
ART regimen						
Nil ARVs	133 (89.86)	15 (10.14)	0.464	1 (ref)	1 (ref)	
AZT/NVP i, ii	901 (92.51)	73 (7.49)		0.72 (0.40–1.29)	0.37 (0.10–1.45)	0.153
D4T/3TC/NVP iii	824 (90.85)	83 (9.15)		0.89 (0.50–1.59)	0.29 (0.08–1.07)	0.063
EFV/TDF/FTC	1533 (92.02)	133 (7.98)		0.77 (0.44–1.35)	0.25 (0.07–0.87)	0.030

*i* Gestational Age (GA), *ii* row percentage, *iii* below 10th percentile, *iv* Chi-square, *v* Odds Ratio, *vi* following variables were adjusted for in the multivariable adjusted model, *year* age group, mode of delivery, HIV status

*i* AZT/NVP vs D4T/3TC/NVP p-value = 0.410, *ii* AZT/NVP vs FTC p-value = 0.521, *iii* D4T/3TC/NVP vs FTC p-value = 0.800, *iv* following variables were adjusted for in the multivariable adjusted model, *year* age group, mode of delivery, HIV status, CD4 (HIV positive mothers only), ART regimen (HIV positive mothers only)

Among HIV infected women only, receiving any ART regimen vs not receiving any ART was associated with significant and highly reduced odds of a preterm birth and low birth weight. Although the antiretroviral regimens appear to confer some protection this was only statistically significant at 5 % level for use of the fixed dose combination (TDF/FTC/EFV) and small for gestational age (GA) outcome. Pairwise comparison of the regimens suggested no significant difference in odds ratios for pre-term delivery, low birth weight or small for GA (*p* > 0.05).

## Discussion

South Africa implemented new ARV guidelines in 2014 with an intention to improve antiretroviral treatment coverage for all HIV positive pregnant women. For each category of birth outcomes we compared the frequency in 2014 with 2011. Overall, we observed a significant improvement in the stillbirth and preterm delivery rate in 2014 and we also observed the larger coverage (94.8 %) of triple antiretroviral treatment in 2014. Although, after adjusting for CD4 category the 2 drug (AZT/sd NVP) was as protective against still birth, low birth weight and preterm deliveries, among the immunocompetent HIV positive women. In a bivariate analysis of all deliveries, unregistered pregnancies and HIV infection remained significant risk factors for still birth, preterm deliveries (<37 weeks), low birth weight (<2500 grams) and small for gestational age. We further found that when compared to HIV negative women, women who did not register for antenatal care and whose HIV status was unknown at the time of delivery were 6.5 times more likely to have a still birth, 4.5 times more likely to have a low birth weight baby (<2500 grams), 3 times more likely to have a preterm delivery (<37 weeks), and 2 times more likely to have a small for gestational age baby (SGA).

Although studies in Europe and North America reported an increase in the preterm delivery rate after the introduction of HAART [17], we have neither seen an increase nor a reduction in preterm deliveries in this population between 2011 and 2014. As we have demonstrated the reduced odds of preterm delivery in association with antiretroviral exposure, it is possible that the high preterm delivery rate among women who did not receive antenatal care in combination with substandard obstetric practice could be reasons for the unaltered preterm delivery rate between 2011 and 2014. Other studies have specifically implicated the use of PI-based antiretroviral regimens in the increased rate of preterm deliveries [12, 18, 19]. None of the women in our analyses were on a PI containing second line regimen, however we did have an opportunity to explore the potential adverse effects of an EFV containing regimen implemented in 2014 in comparison to a NVP containing regimen implemented in 2011. Consistent with findings from other studies in sub Saharan countries, we did not detect any association between still births, preterm delivery and low birth weight with NVP or EFV [20, 21].

Antiretroviral specific associations were reported in a multinational PMTCT study that suggest an increased risk of preterm deliveries (<37 weeks) and LBWR (<2500 g) with a triple ARV PI based regimen as compared to a Zidovudine based dual regimen [16]. The study further reported a higher incidence of more severe birth outcomes (PTD <34w) and neonatal deaths associated with a TDF based triple regimen as compared to the Combivir based triple regimen. Although the frequency of stillbirths, preterm births and low birth weight were higher among women receiving a TDF based regimen when compared to the Stavudine containing regimen, the association was not statistically significant. Although, it must be noted that women who were on D4T/3TC/NVP during 2011 met the criteria for treatment (CD4 < 350) and women receiving TDF/FTC/EFV in 2014 received treatment irrespective of CD4+ count.

Unregistered pregnancies are considered high risk for HIV and adverse obstetric outcomes. It is probable that the majority of the unregistered pregnant women in our study were HIV infected and not received any form of antiretroviral. The women would have been tested for HIV after delivery and the results for which we did not have access. This is one plausible explanation for the high rate of adverse pregnancy outcomes among the unregistered pregnancies in our study. In a study conducted in South Africa in the early years of the epidemic (1999) unregistered pregnant women were 1.4 times more likely to test HIV positive as compared to their registered antenatal counterparts. HIV prevalence in unregistered pregnancies and antenatal population groups were 45.2 (95%CI 37.9–52.5) and 32.5 % respectively [22]. Similar findings were reported for a low HIV prevalence setting in Atlanta 1.4 % vs 0.4 %) [23]. Notably, unregistered pregnant women are also more likely to be younger, single with no partner support, of low socioeconomic status and with minimal education; all of which could likely contribute to adverse birth outcomes and HIV acquisition [24].

An alternative explanation could be that the unregistered pregnant women did not receive the much needed antenatal care which could have prevented the adverse pregnancy outcomes. In a case controlled Nigerian study, unregistered pregnant women were twice as likely to deliver preterm (20.0 vs 10.9), 1.5 times as likely to deliver low-birth weight babies (36.9 vs 25.6) and seven times more likely to have still births (17.7 vs 3.3 %) when compared to registered antenatal attendees [25].

## Conclusions

In summary, when compared to HIV uninfected women, HIV infected women have a higher risk for stillbirth, PTD, SGA, and LBW babies. However ART exposure as ZDV prophylaxis or triple ARV regimen is associated with decreased odds for an adverse birth outcome. Untreated HIV infected women and women who do not seek antenatal care should be considered most at risk for poor birth outcomes.

Our study is not without limitations. Births <500 g were not recorded in the birth register and although this is an adverse obstetric outcome, it was not included in the analysis i.e. potential selection bias. The use of gestational age to define preterm delivery was entirely dependent on a single value recorded in the maternity register. Gestational age at birth is usually extrapolated from antenatal assessments that are not always accurate. Ballard scoring in the absence of an antenatal ultrasound assessment which is more reliable is not routinely used. The major limitation is the lack of information in maternity registers such as duration of ART prior to delivery or whether women initiated ART either during pregnancy or before pregnancy. Information related to socio-economic status are also unavailable. A further limitation is the inability to adjust for other improvements in the obstetric care that may have contributed to the better birth outcomes. However, there was no evidence of a change in management, staff training, additional nursing staff or a change in obstetric management protocols.

## Abbreviations

HIV: Human immunodeficiency virus
AIDS: Acquired immunodeficiency syndrome
ARV: Antiretroviral
ART: Antiretroviral therapy
SSA: Sub-Saharan Africa
FDC: Fixed dose combination
D4T: Stavudine
AZT: Azidothymidine
3TC: Lamivudine
NVP: Nevirapinne
TDF: Tenofovir
FTC: Emtricitabine
EFV: Efavirenz
PI: Protease inhibitor
SB: Still birth
PTD: Preterm delivery
VPTD: Very preterm delivery
LBW: Low birth weight
VLBW: Very low birth weight
GA: Gestational age
SGA: Small for gestational age
MTCT: Mother-to-child transmission
PMTCT: Prevention of mother to child transmission
MDG: Millennium development goals
WHO: World Health Organisation
OR: Odds ratio
